# Lysmeral Exposure in Children and Adolescences Participating in the German Environmental Survey (2012–2015): Integrating Sex/Gender into Analysis

**DOI:** 10.3390/ijerph192417072

**Published:** 2022-12-19

**Authors:** Sophie Ch. Fichter, Katrin Groth, Nina Fiedler, Marike Kolossa-Gehring, Małgorzata Dębiak, on behalf of the INGER Study Group

**Affiliations:** German Environment Agency (UBA), 06844 Dessau-Roßlau, Germany

**Keywords:** lysmeral, butylphenyl methylpropional, lily aldehyde, lilial, human biomonitoring, sex, gender, sex/gender, GerES

## Abstract

Comprehensive consideration of the biological and social diversities of sex and gender as well as their interdependencies is mostly missing in human biomonitoring (HBM) studies. Using the INGER sex/gender concept as theoretical background, we analyzed differences in exposure to lysmeral, a compound commonly found as a fragrance in cosmetics, personal care, and household products, in 2294 children and adolescents in Germany using decision tree, regression, and mediation analysis. The variables “sex assigned at birth” and “age”, as well as well as use of personal care products and fabric conditioner proved to have the highest explanatory value. Mediating effects of behaviour associated with societal gender expectations were observed, as the use of cosmetics correlated highly with lysmeral metabolites concentrations in girls between 6 and 17 years, with the strongest effect in adolescents between 14 and 17 years old. In the youngest age group (3–5 years) boys showed higher concentration of the metabolite tert-butylbenzoic acid (TBBA) compared to girls of the same age but only if TBBA urine concentrations were normalized on creatinine. Our study offers the first retrospective sex/gender assessment of HBM data. It demonstrates the possibilities to rethink and broaden sex/gender analysis in existing HBM-studies and highlights the need for inclusion of new sex/gender concepts in the design of new studies.

## 1. Introduction

Human biomonitoring (HBM) is a tool of health-related environmental monitoring. It includes an assessment of concentrations of chemicals or their metabolites in human matrices, mostly blood or urine. HBM data reflect the total body burden of chemicals accounting for all exposure pathways, individual variability in exposure levels and toxicokinetic. As such, HBM is an important tool for estimating potential health risks linked to the chemical exposure and environmental pollutants. The comparison of HBM data with health-related guide values or statistically derived reference values allows the identification of population at risks [1,2]. We and the others have identified a number of chemicals for which differences in body burden between males and females were observed e.g., male participants had higher concentrations of per- and polyfluoroalkyl substances (PFAS) [3], quicksilver [4], polychlorinated biphenyls (PCBs) and hexachlorobenzene (HCB) [5], glyphosate and AMPA [6], and some polybrominated diphenyl ethers (PBDE) [7] than female participants. The copper levels in blood plasma were in turn substantially higher in females than in males [8]. At the same time various geographical, sociological and behavioral factors were associated with differences in chemical body burden on single compound basis [3,5,7,9,10,11]. In context of children’s chemical exposure, special attention needs to be given to the chemical burden, habits and living environment of the mothers. Firstly, children and their mothers, sharing diets and home environments, also share exposure in common consumer products related chemicals [12]. Secondly, the chemical transfer from mothers to children may appear due to pregnancy and breast feeding, e.g., the PFAS concentrations in children were associated with the age of mothers at birth of the first child and length of breastfeeding [13], and finally, the exposure might be associated with the care taking activities, e.g., urine cotinine concentrations of passive smoking children were higher if mother was smoking in comparison to smoking father [14]. 

Although the examples of sex/gender dependent differences in chemical exposure are growing in number and the theoretical concept linking sex/gender with chemical exposure and body burden has been already established for some time [15,16], the comprehensive consideration of sex/gender in HBM studies is still missing [17]. This is in line with the general trend in environmental health studies [17,18,19] and is at least partly a consequence of missing applicable concepts and tools. 

To overcome this obstacle, the collaborative and interdisciplinary research project on integrating gender into environmental health research (INGER) has brought together gender theoretical background information and designed a model for application in environmental health studies [20]. In general, for humans, the term sex is used to describe biological characteristics such as chromosomes, reproductive/sexual anatomy or hormone levels, while the term gender refers to social factors, both on an individual and a structural level (i.e., individual and societal expectations/gender roles, identities) [21]. In current understanding, these different dimensions, while influencing and interacting with each other, do not necessarily depend on each other and—although often categorized as binary—can exist on a continuum [22,23,24]. In order to illustrate this, we use the term “sex/gender” in the following, unless talking about a specific variable [25,26]. The INGER concept combines four prerequisites for sex/gender analysis: multidimensionality, variety, embodiment, and intersectionality. It encompasses dimensions of the individual sex/gender self-concept (sex assigned at birth, phenotype at birth, current sex/gender identity, current phenotype, internalized gender roles, and externalized gender expressions) as well as items contributing to explain structural sex/gender relations (such as education, income, family situation, and ethnicity) as well as psychosocial factors such as smoking and dietary habits or stress (for the whole list see [20]. In this work, we applied the INGER sex/gender concept for the first time for a retrospective data analysis of the 5th German Environmental Survey for children and adolescents (GerES V), using a model substance—lysmeral—as an example. GerES V is a cross-sectional human biomonitoring (HBM) study involving 2291 participants aged 3–17 years. It collects extensive information on health- and exposure-relevant behaviours via self-reported questionnaires and interviews [27]. So far, these questionnaires have not been assessed on the dimensions of sex/gender and sex/gender has not been implemented in the analysis of GerES V beyond a routine male/female stratification. 

Lysmeral (2-(4-tert-butylbenzyl)propionaldehyde) is a synthetically produced aliphatic-aromatic aldehyde reminiscent of the smell of lily of the valley. In the international nomenclature for cosmetic ingredients (INCI), it is listed under the name butylphenyl methylpropional, and is widely used in cosmetics, personal care products and household products. The predominant exposure route is dermal, but lysmeral can also be inhaled or taken orally. The Risk Assessment Committee (RAC) has rated it as reprotoxic. In addition, lysmeral is suspected of being skin sensitizing and having a hormone-disrupting effect. Differences in chemical body burden of male and female participants as well as positive associations of use of personal care products, fragrances and fabric softener with urinary concentrations of lysmeral metabolites have been shown previously [28]. Based on these first descriptive analyses, we used the INGER sex/gender concept to investigate the observed effects related to the binary sex/gender stratification in more detail. It is the first application of theoretical sex/gender concepts for retrospective analysis of HBM data with the aim of testing of available methodology and drawing the recommendations for the implementation of sex/gender HBM studies.

## 2. Materials and Methods

### 2.1. Study Design and Sample

The 5th German Environmental Survey for children and adolescents (GerES V) is a cross-sectional human biomonitoring (HBM) study involving 2291 participants aged 3–17 years. It focuses on human exposure to environmental chemicals and its sources and collects blood and urine samples for analysis of chemical body burden as well as extensive information on health- and exposure relevant behaviours via self-reported questionnaires and interviews.

The GerES V population is a stratified random subsample of the German Health Interview and Examination Survey for Children and Adolescents (KiGGS Wave 2) of the Robert Koch Institute (RKI) [29]. From January 2015 to June 2017, a sample was recruited as representative of their age and sex based on population registry data in a random two-stage sampling procedure. Briefly, RKI recruited a population representative sample in 167 locations in Germany reflecting the grade of urbanization and geographical distribution which are considered representative of the whole of Germany. Out of these, the 3–17 years old random subsamples were drawn [29]. The study population characteristic is described by Schulz et al. [30].

The concentration of various substances and their metabolites were determined in blood or urine of participants depending on the chemical characteristics. Questionnaires were used to obtain information on exposure relevant conditions, habits, and behaviors of the participants. Parents or legal guardians of the participants and the participants themselves (if aged 11 or older) filled up self-assessment questionnaires and were additionally surveyed by an interviewer during a home visit.

Participation in the study was voluntary. All participants or their legal guardians were informed about the aims and contents of the study as well as about data protection and gave their informed consent. The study was approved by the Ethics Committee of the Berlin Chamber of Physicians (Eth-14/14).

### 2.2. Chemical Analysis

Lysmeral metabolites were determined in urine with the method by Pluym et al. [31]. This method allows the parallel determination of the parent compound and its metabolites (4-tert-butylbenzoic acid (TBBA), lysmerol, lysmerylic acid, and hydroxylated lysmerylic acid). Creatinine content of the urine samples was photometrically determined by a validated standard assay based on the Jaff’e method.

### 2.3. Variable Selection for the Retrospective Analysis of Sex/Gender Impact on Lysmeral Body Burden

All available variables of the GerES V questionnaires were reviewed for their suitability to operationalize the INGER sex/gender concept. Implementing multidimensionality, variety, embodiment and intersectionality, the INGER sex/gender concept can be used to systematically review existing variables in order to expand sex/gender-related information. It differentiates between dimensions of the individual sex/gender self-concept (sex phenotype at birth/sex assigned at birth; current sex phenotype; current sex/gender identity; internalized sex/gender roles; externalized sex/gender expressions), items contributing to explain structural sex/gender relations (such as experiences of discrimination and care activities as well as intersectionality-related social categories) and incorporates sex/gender-related lifestyle and psychosocial factors (such as smoking and dietary habits and stress perception) [20]. All variables of GerES V that were thus identified were selected for further analysis and can be found in Table S1.

### 2.4. Statistical Analyses

Analyses were conducted for volume-based as well as creatinine-adjusted concentrations. Creatinine adjustment serves the purpose of adjusting for differences in filtration rate and is also affected by factors like diet, fluid intake and body composition. All statistical analyses were performed with R (version 4.1.0). Two different decision tree variants were applied on all analyses: CART (Classification and Regression Tree, [32], implemented in the rpart package [33], and CIT (Conditional Inference Trees, [34]), implemented in the partykit package [35]. Stopping criteria for CART trees was set at a minimum number of observations of n = 50 and a maximum tree depth of 4. In addition, the significance level for the CIT trees was set to *p* = 0.05 (adjusted for multiple comparisons using Bonferroni correction). 

As per decision tree mechanics, all covariates were included into the analysis at once, with one analysis each for TBBA and lysmerol, with and without creatinine adjustment. 

In order to examine intersectional sex/gender interactions we included the relevant factors “age group” and “socioeconomic status” in a comparison of means between groups. Significance was determined by Welch two-samples *t*-test.

Multiple linear regression models were calculated using case weights provided by the RKI [36], adjusting to match the population structure of the 3–17-year-olds in Germany. All relevant variables identified in the decision tree analysis were included in the multiple linear regression. Dummy variables were created for nominal and ordinal data. Models were calculated using the backward deletion process.

In order to further deepen the understanding behind significant differences based on “sex as-signed at birth”, model-based causal mediation analysis was done using the mediation package [37]. Variable combinations examined were the correlation between “sex assigned at birth” and “socioeconomic status” with metabolite concentration, mediated by “use of fabric softener”, “use of perfume” and “use of eye make-up”.

A moderated multiple regression (MMR) was calculated in order to determine whether the relationship between the use of various consumer products was influenced by sex assigned at birth [38].

## 3. Results

### 3.1. Variable Selection

Of the GerES V-variables, 119 were identified as potentially relevant based on the INGER sex/gender concept. The majority of these variables concerned items contributing to explaining structural sex/gender relations as well as lifestyle and health-related variables. A total of 40 of 119 variables were related to perceived stress, emotional situation, quality under consideration of the living situation and information about inattention/hyperactivity in children as well as emotional wellbeing (e.g., “Are you satisfied with yourself?”). Social support was determined with nine questions, such as “Do you have someone who listens to you?”. Experiences of discrimination were determined in 14 questions; ethnicity in 15. Information on religious affiliation and sexual orientation information was deducted from the question “Do you experience discrimination based on …?”. Education, occupation, employment, income, and social status were documented with 23 variables. Another 10 variables provide selected information about diet type, smoking and physical exercise. Eight biology-related variables (weight, height, body surface, BMI) were also considered. 

Information regarding the individual sex/gender self-concept was collected only by the single ambiguous question “What sex/gender does your child have?” (“Welches Geschlecht hat Ihr Kind?”) or “What is your sex/gender?” (“Was ist dein Geschlecht?”, for children older than 14) with binary response options being “boy” or “girl”. As this information was based on and compared with official census/registry data we retrospectively assumed that this variable is consistent with “sex assigned at birth”, which we will use in this publication. The “intersex” option for sex assigned at birth was only established in 2018, after completion of the survey period and does thus not appear. Questions about gender identity, gender expression and gender roles were not part of the GerESV questionnaire, and neither were specific questions about biological dimensions of sex (such as chromosomes, hormonal status, or sexual organs). 

Based on the known applications of lysmeral, 16 variables for potential exposure sources were included into analysis in order to put them into relation with the selected sex/gender variables. All in all, a set of 141 variables was subjected to the decision tree analysis.

A full list of variables can be found in the Supplementary Materials (Table S1).

### 3.2. Decision Tree Analysis for Variable Exploration and Reduction

As a next step, we used decision trees to investigate the relevance of the selected variables for lysmeral body burden (Table 1). All covariates were included into the analysis at once, with one analysis each for TBBA and lysmerol, for both volume-based concentrations as well as creatinine-adjusted. 

**Figure 1:**
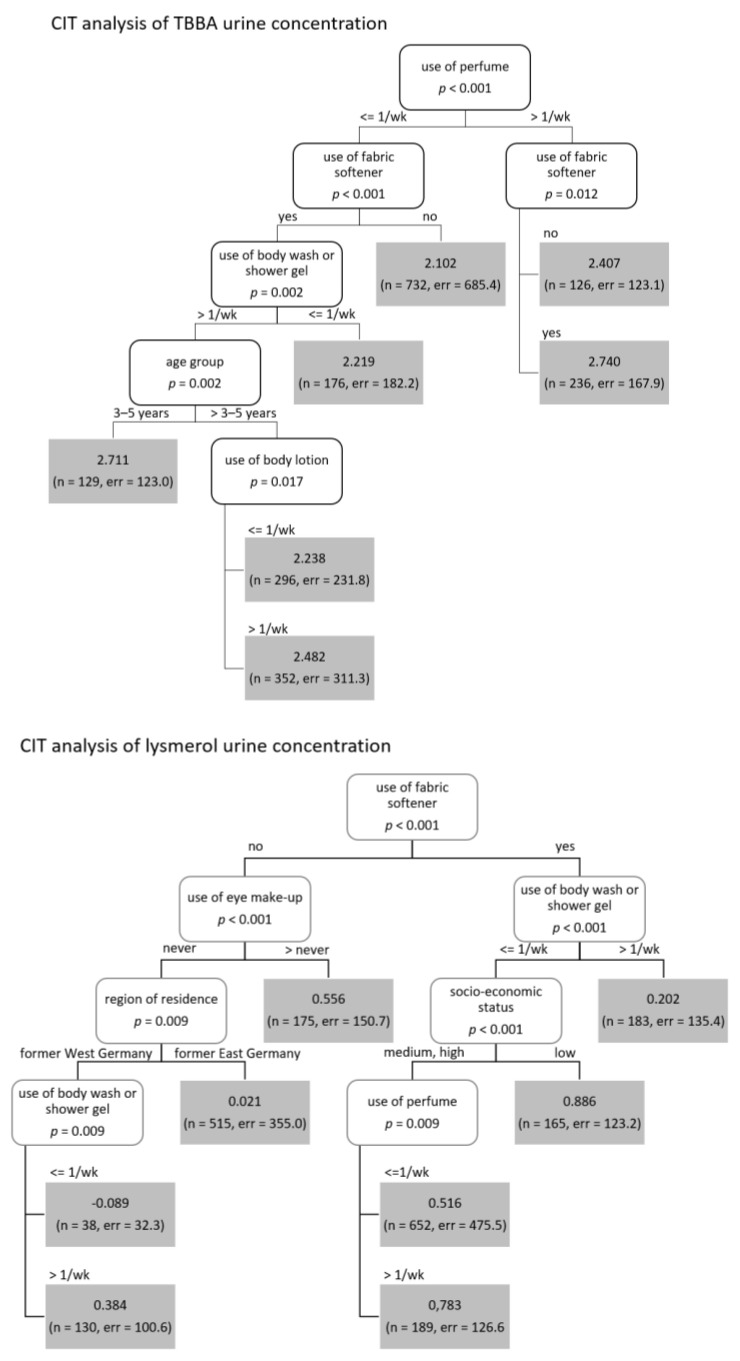
CIT results for volume-based TBBA and lysmerol urine concentrations for all participants.

**Figure 2:**
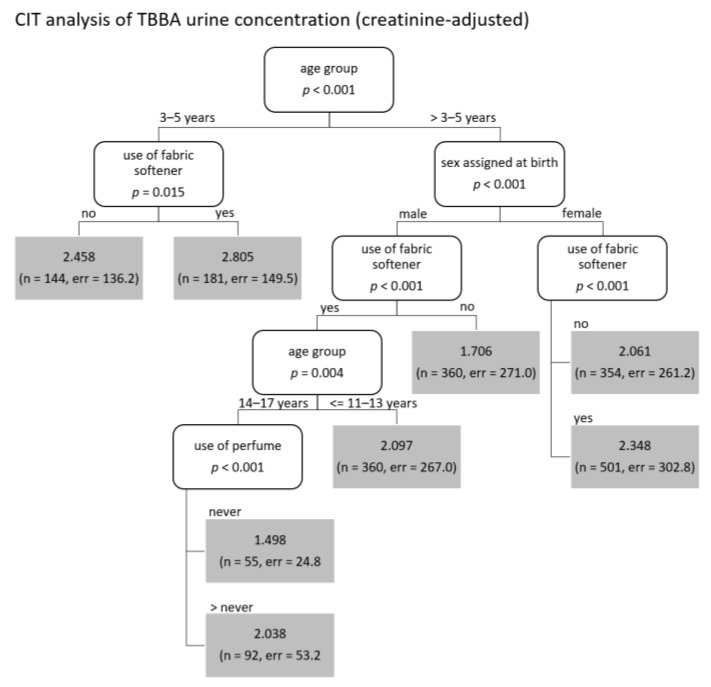

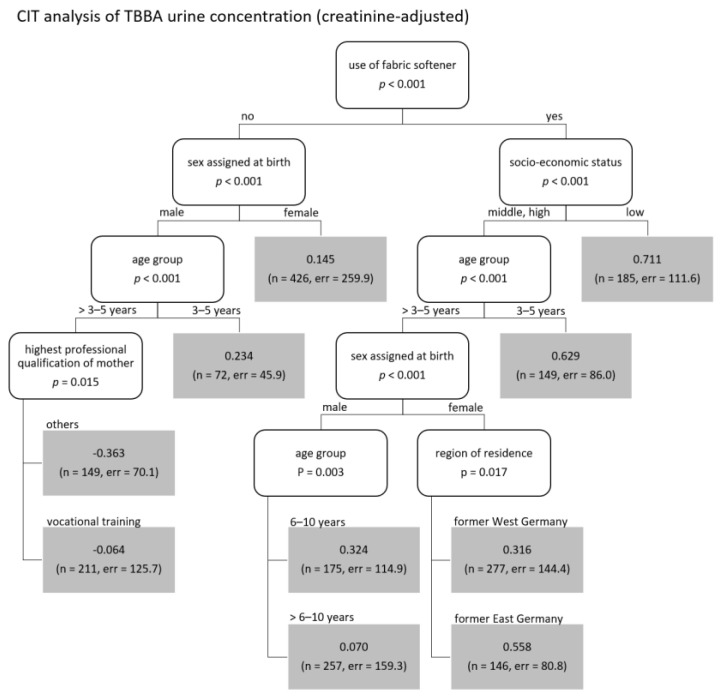
CIT results for creatinine-adjusted TBBA and lysmerol urine concentrations for all participants.

Overall and regardless of decision tree specifics, the analyses supported the study Murawski et al., (2020). Table 1 shows all covariates selected by the decision trees, which are identical to the ones identified in the previous study. For creatinine-adjusted lysmerol urine concentration, factors such as “age”, “sex assigned at birth”, “socioeconomic status” and “east/west Germany” appear more often and add more explanatory value than for volume-based TBBA/lysmerol concentrations, where exposure sources show higher impact (Figure 1 and Figure 2). However, the most prominent variable is the “use of fabric softener”, which appeared as the first node in all trees regarding lysmerol concentrations as well as volume-based TBBA. For creatinine-adjusted TBBA concentrations, “use of fabric softener” appears as second and third node, respectively, with “age group” as most important variable and “sex assigned at birth” as second most important variable for all age groups older than five years. “Age group” adds explanatory value to three trees, “sex assigned at birth” only appears in creatinine-adjusted trees. Exposition sources “use of perfume” and “use of body wash/shower gel” appear in three and four trees, respectively, but more prominently in trees for volume-based metabolites concentrations. “Use of eye-make-up”, while being highly explanatory in volume-based trees (second node), does not appear in trees for creatinine-adjusted metabolites concentrations at all.

In order to narrow down factors, decision tree analysis was repeated and stratified by four age groups: 3–5 years old, 6–10 years old, 11–13 years old, and 14–17 years old. In general, the number of relevant covariates decrease for age-separated analyses compared to all-ages analysis, with four nodes being the maximum number of covariates present. For the youngest age group, “use of fabric softener” is again the first relevant variable in all eight trees and the only relevant variable in four of them. The other two identified variables for 3–5-year-olds are “use of perfume” and “East/West Germany”. For 6–10-year-olds as well as 11–13-year-olds, “use of body wash/shower gel” appear as an additional relevant variable in volume-based trees, “socio-economic status” and “sex assigned at birth” (respectively) in creatinine-adjusted trees. For the oldest age group, “use of perfume” replaces “use of fabric softener” as most descriptive variable in all trees (first node) and “use of eye-make-up” is added as a factor to TBBA trees. Lysmerol body burden, independent of creatinine-adjustment, is described with “use of perfume”, “socio-economic status”, “sex/gender” and “use of fabric softener”, while TBBA body burden with “use of perfume”, “use of eye-make-up” and “use of fabric softener”. 

All in all, the analysis shows that exposure routes as well as socio-economic factors can be used to describe lysmeral metabolite body burden, depending on which metabolite was examined and whether creatinine adjustment was considered. In general, “use of fabric softener” seems to be a useful indicator in most cases, although it is replaced by other consumer products (especially “use of perfume”) for older children and teenagers. Creatinine adjustment leads to a higher relevance of factors such as “sex assigned at birth”, “East/West Germany” and “socioeconomic status”, while volume-based body burden seems to have a stronger connection to exposure sources. However, for the oldest age group, this distinction is between TBBA (exposure routes) and lysmerol (social-economic factors + exposure routes). Distinction of age groups narrows the information down further, as lysmeral sources varies between ages. “Use of eye-make-up” only appears in TBBA trees.

### 3.3. Lysmerol Metabolite Body Burden Depending on “Sex Assigned at Birth”, “Age Group” and “Socioeconomic Status”

**Figure 3:**
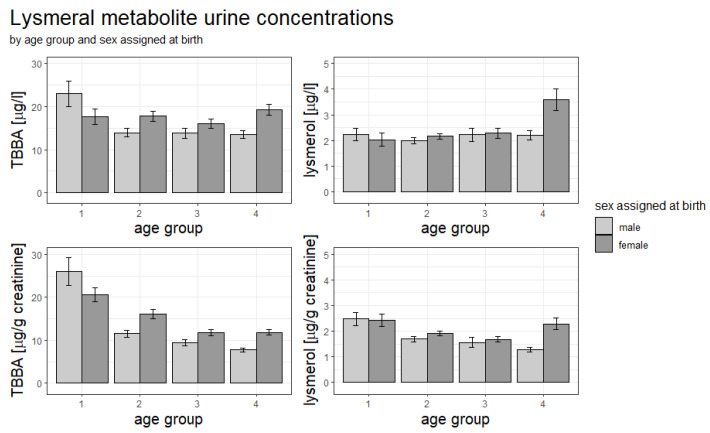
Concentrations of lysmeral metabolites tert-butylbenzoic acid (TBBA) and lysmerol, both volume-based and creatinine-adjusted, grouped by age group and sex assigned at birth. Bars represent standard deviations. Age groups are 3–5 years (1), 6–10 years (2), 11–13 years (3) and 14–17 years (4).

Significant differences in metabolite concentrations in subgroups determined by “sex assigned at birth”, “age group” and “socioeconomic status” were shown previously (Murawski et al., 2020), with female participants showing a significantly higher metabolite urine concentration than male participants. Looking at mean comparisons using the Welch *t*-test, “sex assigned at birth” and “age group”, however, lysmerol concentration was only significantly different between boys and girls for the oldest age group while TBBA showed significant differences in metabolite concentration between boys and girls for three (volume-based) and all four (creatinine-adjusted) age groups (Figure 3). The differences in TBBA urine concentration between boys and girls were especially of interest, as for the youngest age group, boys show a higher TBBA concentration (both for volume-based and creatinine-adjusted values) while for older children and adolescents the ratio flips. With creatinine-adjusted data, younger age groups show higher relative concentrations than older age groups for both TBBA and lysmerol concentrations. 

Concerning the socio-economic status, the univariable analysis shows significantly higher metabolite urine concentration for lower status groups, with differences and significance being more pronounced for lysmerol. This could be confirmed in the multivariable analysis. Looking at both age group and socioeconomic status, the difference in lysmerol urine concentration between participants with lower socioeconomic status and those with medium and high socioeconomic status is distinctly more pronounced for the youngest and oldest age group.

Analysis of a combined socio-economic status and sex assigned at birth also showed significantly higher lysmerol urine concentrations for groups with a low socio-economic status for both boys and girls, with girls showing significantly higher concentrations than boys. These differences between men and women decrease for groups with a medium socio-economic status and vanish for groups with a high socio-economic background along with the general lysmerol urine concentration. 

### 3.4. Multiple Linear Regression Analysis 

Multiple linear regression models were calculated for all outcomes and included all significant variables from the decision tree analysis. Explained variance of volume-based metabolite urine concentration was 23% for TBBA and 26% for lysmerol. Explained variance for age group models varied but were all in all the highest for 14–17-year-olds (TBBA 32%) and 3–5-year-olds (lysmerol 33%). 

Explained variance for creatinine-adjusted metabolite concentration was lower with 15% for TBBA and 14% for lysmerol. Models for the 14–17-year-olds explained 17% of variance for both TBBA and lysmerol. 

**Figure 4:**
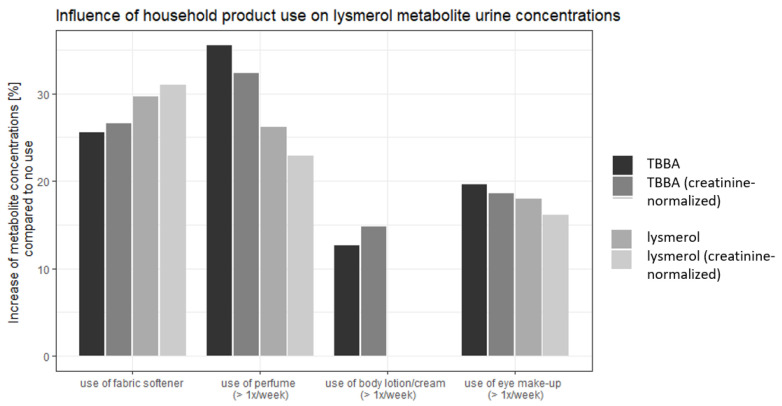
Results of multivariable regression: Increase in metabolite urine concentrations for use of household products compared to no use.

Effects of use of consumer products were consistent between models (Figure 4): use of fabric softener was associated with an increase of all four metabolites urine concentrations by 26% to 31%, while use of perfume more often than once a week led to an increase of 22% to 32% if compared to the participants who used these products less often or not at all. Use of eye make-up (more often than once a week) increased concentrations by 16% to 19%. Statistically, female participants’ urine metabolite concentrations were 10% to 20% higher than males’. Participants with medium and higher socioeconomic status showed 20% and 30% higher metabolites’ concentrations than participants with a low socio-economic status, respectively. 

Distinguished by age, different exposure sources were definitive for the lysmeral body burden: for the youngest age group, use of fabric softener led to the highest increase in metabolite concentration (43–47%), whereas for the oldest age group, it was the use of perfume (47–50%). Female participants showed 36–38% increase in lysmerol metabolite concentration. All results of multiple linear regression model analyses can be found in the Supplementary Materials.

### 3.5. Mediation Analysis

Mediation analysis was performed for all participants as well as age-groups separated in order to further examine the relationship between the factor “sex assigned at birth” and lysmeral metabolite urine concentration using the use of consumer products (fabric softener, perfume and eye make-up) as mediators. For ages 3–13 years, no mediation could be detected. For the oldest age group, the effect of “sex assigned at birth” on metabolite urine concentration was mediated in part by “use of perfume” (37%) and “use of eye-make-up” (14%). The of use of fabric softener was the same for girls and boys of the same age. 

## 4. Discussion

The aim of this study was the retrospective analysis of human biomonitoring data using a new thought basis for exploring sex/gender differences in lysmeral exposure. The results of the univariable analysis have shown a positive association between urinary concentrations of lysmeral metabolites and use of personal care products, perfumes and fabric softener [28]. This corresponds to authorized use of the compound in consumer products according to the REACH Regulation (EC 1907/2006) [39]. In addition, girls and participants with a lower socio-economic status had higher metabolites urine concentrations than boys and participants with higher socio-economic status. 

To investigate the sex/gender impact on the lysmeral body burden in more detail and to identify new exposure relevant factors, we performed a multivariable analysis of lysmeral metabolite concentrations with additional sex/gender related variables using decision trees as well as regression analysis.

One possibility to retrospectively include more sex/gender dimensions in environmental health analysis is the construction of gender indices. For this purpose, available sex/gender-related variables are analysed by means of regression models with biological “sex” as a dependent variable to calculate the gender index [40,41,42]. While the merging of variables into one index is a statistically efficient option, this approach has also drawbacks such as the need of calculation of the index for each single study, lack of standardized criteria for variable inclusion, differences in the information content used for calculation, and the strong reference to the binary male/female, undefined “sex”, mostly used as a bipolar scale, reinforces binary structure and stereotypes [18,20].

Therefore, we have decided to follow an alternative approach where all available sex/gender related variables according to the INGER sex/gender concept were included in the multivariable analysis. The variable selection based on INGER concept has shown that the GerES V questionnaires cover a wide range of intersectionality-related variables as well as variables related to structural sex/gender relations. Consolidated and used purposefully, this information can be used for advanced sex/gender analysis and intersectional approaches. However, important aspects of the INGER sex/gender concept were not addressed in GerES V, in particular concerning the individual sex/gender self-concept as the study includes only a binary sex assessment. 

In GerES V, sex/gender (“Geschlecht”, meant as biological sex, male/female) has only been determined with one question: “What sex/gender does your child have?” This is doubly ambiguous as the German language mainly uses the same word for “sex” and “gender” and the questionnaire does neither specify how the sex should be assessed, nor differentiate between sex assigned at birth and current sex/gender identity. The binary sex assessment was a common standard in environmental health studies. However, it was critically evaluated in gender transformative environmental health studies [43,44]. 

For GerES V children were recruited using official census/registry data, following by the interview. Based on concordance of these data, we assume that the sex/gender-variable as collected in GerES V is consistent with “sex assigned at birth”. This variable is the only information related to the individual sex/gender self-concept, as questions about gender identity, (specified) biological sex, gender expression and gender roles are not part of the GerESV questionnaire. Consequently, central sex/gender dimensions are missing in our analysis. It corresponds with current standards of HBM questionnaires. Recently, researchers and authorities have stressed the importance of incorporating more sex/gender dimensions in all phases of research as well as promoting a more inclusive and varied sex/gender understanding into environmental health studies in general [20,41,43,45]. 

Although our study shows that the INGER sex/gender concept was a useful tool for a retrospective data analysis, for the integrated sex/gender implementation in HBM studies, a consideration of sex/gender at the planning phase of the studies is necessary. The INGER sex/gender concept in combination with other instruments might be very beneficial at this stage for the identification of the variables, questionnaire design, and planning of analysis strategy. The Gender and Chemicals Roadmap [16] offers a broader overview as well as practical impulses for the systematic integration of sex/gender in the field of chemical management. 

In the second part of this study we performed a multivariate analysis of the available sex/gender variables in order to identify previously undetected relations with chemical exposure. To prevent assumptions from influencing the selection of covariates for the statistical model, we used decision trees, which have been successfully used in health research, for example in epidemiology [46], mental health [47], and public health [48,49]. As they do not have any presumptions about the data at all, they can be used for analyses which have to deal with many covariates and data structures and can detect non-linear and complex correlations. In our case, both CART and CIT trees handled the data easily and gave similar results. Newer studies suggest that multilevel analysis of individual heterogeneity and discriminatory accuracy (MAIHDA) might be considered for further analysis, as it deals especially well with many covariates and relatively small number of samples [50].

As no new variables proved to be statistically relevant we fell back on using “sex assigned at birth” as an analytic variable. We are aware that this perpetuates binary stereotypes, however, the binary assignment as a social structure category has a high explanatory power [44]. However, it has to be kept in mind that the results reflect only correlations and not causalities.

According to the analysis in Murawski et al., age and sex were the most significant influence factors with girls being the highest-exposed group. Our study confirms and differentiates results from Murawski et al. [28]. The main additional explanatory value lies in the identification of sex/gender-dependent exposure mechanisms and identification of the additional highly exposed group of the young children. Moreover, by use of multivariable analysis, we were able to show that higher exposure of girls is in part determined by use of cosmetics. Closer examination of exposure pathways is still needed to determine the rest of the differences. Differentiation by age revealed that young children had as high concentration of lymerol in urine or even higher concentration of TBBA than teenaged girls. Lysmeral metabolism has been investigated in rodents and humans after oral exposure. In humans the excreted metabolites of lysmeral are clearly dominated by the secondary metabolite TBBA, which represented on average 14.3% of the administered dose, followed by the primary metabolite lysmerol, yielding 1.82% of the dose after 48 h. Further metabolites hydroxy-lysmerylic acid and lysmerylic acid represent only 0.20 and 0.16% of the dose, respectively. While lysmerol is its structural similarity regarded as specific metabolite of lysmeral, TBBA has been reported to be also a product of 4-tert-butyl-toluene metabolism [51]. The analysis of samples of Environmental Specimen Bank showed a high correlation between TBBA and lysmerol urine concentrations (r = 0.577; *p* < 0.001), confirming that both metabolites are suitable biomarkers for lysmeral exposure at least for adults [48]. There is no data on lysmeral metabolism in children. In pharmaceutical context however differences between children and adults have been well documented [52]. According to the European Chemical Agency database, TBBA and 4-tert-butyl-toluene are not intended to be used in consumer products. TBBA is used in polymer production as a process regulator (chain stop agent) and as a thermal stabilizer in PVC [51]. The chemical 4-tert-butyltoluene is an intermediate for organic synthesis perfumes and fragrances, among the others, lysmeral. Therefore, the environmental exposure of children with these compounds cannot be fully excluded. 

The differences—higher exposure for younger children compared to older and higher exposure for older girls than older boys— become more pronounced with creatinine adjusted metabolite urine concentrations. Creatinine adjustment serves the purpose of adjusting for differences in filtration rate and is also affected by factors like diet, fluid intake and body composition. Although we are aware of its limitations, in absence of availability to take 24-h-urine samples, we found that creatinine normalization was the best available method to account for differences in urinary filtration rate in GerES V [53,54]. A timeline study with data from the Environmental Specimen Bank showed no significant differences of lysmeral metabolite concentrations between male and female participants in 24 h urine samples for non-creatinine-adjusted data, but did find significantly higher values for women in creatinine adjusted data [48]. Sex- and age-specific physiologically based pharmacokinetic models (PBPK) might provide the additional insights to mechanistic explanations of these sex/gender differences. The pediatric PBPK models have currently reached a sufficient level of maturity for successful application in studies of environmental chemicals [52,55]. 

Knowing the shortcomings of creatinine normalization, our findings are still consistent with current knowledge about children’s vulnerability to environmental chemical exposure. As lysmeral’s main exposure route is dermal, children’s comparatively thin skin and high body surface might contribute to a higher lysmeral intake. The GerES V-questionnaire also does not offer the possibility to assess the quantity of consumer products used as the frequency of use has not been collected. Therefore, there might also be the possibility that parents of younger children use more fabric softener than parents of older children. The other speculation is the relevant inhalative exposure of younger children. 

Higher TBBA urine concentrations found in young boys (especially for creatinine-adjusted TBBA) also cannot be explained with the available data. Lysmeral metabolism has so far only been investigated in adults and the small number of subjects (N = 5) does not allow sex stratification (Scherer et al., 2017). Thus, as we were no able to detect any differences in use of lysmeral containing consumer-product between parents of boys and girls in the GerESV-data; investigation of biological sex/gender factors should be included in further research. 

## 5. Conclusions

- We were not able to fully implement all considerations inherent in the INGER sex/gender concept due to the lack of variables, especially concerning the individual sex/gender self-concept. We did successfully use available data, multivariable analysis as well as mediation and moderation analysis to investigate lysmeral exposure in children and adolescents.
- Sex assigned at birth, age, and use of cosmetics, aromatics and fabric softener proved to have the highest explanatory value for lysmeral exposure. Children aged 3–5 years showed the highest urine concentrations of lysmeral metabolites, regardless of sex assigned at birth. Older children and adolescents showed an increased distinction between boys and girls, with girls showing a higher lysmeral body burden. This increase is highly associated with the use of cosmetics in girls between 6 and 17 years, with strongest effects in adolescents between 14 and 17 years.
- Correlations between “sex assigned at birth” and lysmeral exposure were partially mediated by “Use of Eye Make Up” and “Use of Perfume”, highlighting the impact of behavior associated with societal gender expectations.
- Consideration of sex/gender at the planning stage of the HBM studies is necessary for its sufficient implantation in study design. Approaches for finding a balance between gender theoretical-science and applied environmental monitoring studies need further development.

## Figures and Tables

**Table 1:** Variables selected as most important during decision tree analyses and used in regression analysis. Variables marked with * proved relevant only for lysmerol.

INGER Facet	Variable Name	Coding	N	Missing
Individual sex/gender self-concept				
Sex assigned at birth	Sex assigned at birth	Male	1129	0 (0.00%)
		Female	1165	
Intersectionality-related social categories				
Social Status	SES total score (GerES)	low middle high	268 1366 621	39 (1.70%)
Age	Age group	3 to 5 years 6 to 10 years 11 to 13 years 14 to 17 years	424 752 543 575	0 (0.00%)
Migration	Migration background *	none One-sided Two-sided	1799 214 245	36 (1.57%)
Exposition				
	Use of fabric softeners	no yes	946 1344	4 (0.17%)
	Use of fragrances	no yes	1074 1220	0 (0.00%)
	Use of body wash/shower gel	none once per week or lessmore than once per week	38 391 1864	1 (0.04%)
	Use of body lotions and creams	none less than once per week approximately once per week more than once per week	559 439 382 913	1 (0.04%)
	Use of deodorant	none once per week or less more than once per week	1184 216 893	1 (0.04%)
	Use of perfume	none once per week or less more than once per week	1438 449 404	3 (0.13%)
	Use of eye make up	none once per week or less more than once per week	1839 170 282	3 (0.13%)
Standard variables of GerES V (if not covered under previous dimensions, Murawski et al., 2020)				
	Region of Residence (East Germany/West Germany)	West (incl. West Berlin) East (incl. East-Berlin)	1595 699	0 (0.00%)

## Data Availability

The data presented in this study are available on request from the corresponding author. The data are not publicly available due to privacy reasons.

## Supplementary Materials

The following supporting information can be downloaded at: https://www.mdpi.com/article/10.3390/ijerph192417072/s1, Figure S1: CIT analysis of metabolites urine concentration in 3–5-year-olds; Figure S2: CIT analysis of metabolites urine concentration in 14–17-year-olds; Table S1: Variables considered for extended sex/gender analysis; Table S2: Results of the logistic regression analysis for the urinary TBBA and lysmerol concentration of GerES V participants.
