# Supplementary material for: Lysmeral Exposure in Children and Adolescences Participating in the German Environmental Survey (2012–2015): Integrating Sex/Gender into Analysis
Source: Int J Environ Res Public Health. 2022 Dec 19;19(24):17072. doi: 10.3390/ijerph192417072 (PMC9778794; doi:10.3390/ijerph192417072)
Supplement: Supplementary file 1 [file ijerph-19-17072-s001.zip › ijerph-1974784-supplementary.pdf]

Supplementary to:

Lysmeral Exposure in Children and Adolescences Participating in the German Environmental Survey (2012-2015): Integrating Sex/Gender into Analysis

Authors

Sophie Fichter, Katrin Groth, Nina Fiedler, Marike Kolossa-Gehring, Małgorzata Dębiak*
on behalf of the INGER study group

German Environment Agency (UBA), Berlin/Dessau-Roßlau, Germany

*Corresponding author:
Małgorzata Dębiak
German Environment Agency (UBA)
Corrensplatz 1
14195 Berlin, Germany
Tel.: 0049-30-8903-1217
E-mail: malgorzata.debiak@uba.de

Supplementary to: Lysmeral exposure in Children and Adolescences participating in German Environmental Survey (2012-2015): Operationalization of Sex/Gender

Table S1 ***Variables considered for extended sex/gender analysis***

Facet	Variable name
**Individual sex/gender self concept**	
Sex assigned at birth	Sex assigned at birth
Phenotype at birth	NA
current sex phenotype	NA
current sex/gender identity	NA
internalised sex/gender roles	NA
externalised sex/gender expressions	NA
**Items contributing to explain structural sex/gender relations**	
Income	Monthly net income
Social Status	SES-Subscore: occupational status (GerES)
	SES-Subscore: education/training (GerES)
	SES-Subscore: income (GerES)
	SES total score (GerES)
Family situation	Family situation: With whom does the child mainly live? (GerES)
	Number of persons per household (GerES)
	Only child (GerES)
	Permanent partner: mother/father (GerES)
	Cohabitation with permanent partner of mother/father (GerES)
	Civil status (mother/father) (GerES)
	Scale value family ties
Disability	Officially recognized disability
	Degree of disablement categorized (GerES/KFB)
Sexual Orientation	*Derived from variable Discrimination: Sexual Orientation*
Religion	*Derived from variable Discrimination: Regligion/Faith*
Ethnicity	Migration Status
	Migrant Group (mother) (GerES; KiGGS0 + KiGGS2)
	Migrant Group (father) (GerES; KiGGS0 + KiGGS2)
	Length of residency (parents)
	Length of residency (parents) kat.
	Country of Origin
	Country of Origin (by Region)
	Immigration Generation
	Residential Status (parents)
	Region of Birth (UN)
	Region of Birth (WHO)
	Region of Birth (Destatis)
	Region of Origin (UN)
	Region of Origin (WHO)

	Region of Origin (Destatis)
Child's Education	Which school (GerES)<br>Highest degree of child (expected)
Expectations of child's education	Aspired level of degree (school)<br>Which school performance do you expect from your child? (passing) (GerES)<br>Which school performance do you expect from your child? (effort) (GerES)<br>Which school performance do you expect from your child? (learning targets) (GerES)
Education of parents	Highest school degree (mother) (GerES)<br>Highest school degree (father) (GerES)
Occupation	Current main occupation (coded)<br>Highest professional qualification (mother) (GerES)<br>Highest professional qualification (father) (GerES)<br>Professional Position (mother) (GerES)<br>Professional Position (father) (GerES)<br>Employment (mother) (GerES)<br>Employment (father) (GerES)
Employment	Degree of Employment (mother) (GerES)<br>Degree of Employment (father) (GerES)<br>Current main occupation (coded)
Care activities	Housekeeping load (GerES)

**Lifestyle and Psychological Factors**	
Perception of stress	Financial Worries (GerES)<br>Strain b/c care work in the family (GerES)<br>Strain b/c sole responsibility parenting (GerES)<br>Strain b/c sick family members/ family members in need of care (GerES)<br>Strain b/c conflicts with children/problems with raising children (GerES)<br>Strain b/c conflicts with (former) partners<br>Strain b/c other family members (GerES)<br>Strain b/c living alone/loneliness (GerES)<br>Strain b/c employment situation or unemployment (GerES)<br>Strain b/c little appreciation of house/family work (GerES)<br>Strain b/c chronically ill child/child with disability (GerES)<br>Strain b/c difficulties with compatibility work and family (GerES)<br>Overall strain in everyday life (GerES)
Parents on emotional situation	SDQ emotional problems, German standardization (GerES)<br>SDQ behavioral problems, German standardization (GerES)<br>SDQ inattention/hyperactivity, German standardization (GerES)<br>SDQ Peer problems, German standardization (GerES)<br>SDQ pro-social behavior, German standardization (GerES)

	SDQ total problem score, German standardization (GerES)
Parents on difficulties	SDQ impact score (GerES)
	SDQ impact score categorized (GerES)
	SDQ impact score – high-risk group (GerES)
	SDQ internalizing problems (GerES) - Cutoff (analog Haller et al 2016)
	SDQ externalizing problems (GerES) - Cutoff (analog Haller et al 2016)
Emotional difficulties	Scale value SDQ emotional difficulties (GerES)
	SDQ emotional difficulties, German standardization (GerES)
	SDQ behavioural problems, German standardization (GerES)
	SDQ inattention/hyperactivity, German standardization (GerES)
	SDQ peer-Problems, German standardization (GerES)
	SDQ pro-social behavior, German standardization (GerES)
	SDQ total problem score, German standardization (GerES)
Quality of Life	Quality of Life: feeling well
	Quality of Life: energy
	Quality of Life: sadness
	Quality of Life: loneliness
	Quality of Life: time
	Quality of Life: leisure time
	Quality of Life: being treated fairly
	Quality of Life: fun with friends
	Quality of Life: school
	Quality of Life: concentration
	Quality of Life: exercise
	Quality of Life: running
	Quality of Life: liking life
	Quality of Life: being in good mood
	Quality of Life: fun
	Quality of Life: feeling bad
	Quality of Life: being satisfied with oneself
	Quality of Life: time with mother/father
	Quality of Life: talking to mother/father
	Quality of Life: money to spend with friends
	Quality of Life: money to spend on things
	Quality of Life: time with friends
	Quality of Life: friends helping each other
	Quality of Life: being able to rely on friends
	Quality of Life: happy at school
	Quality of Life: teachers
Body Perception	Body image (GerES)
	Self-image
Diet	Vegetarian

Children/adolescences on social support	Someone who listens
	Someone who shows love/affection
	Someone for having fun with
	Someone who offers information
	Someone who hugs you
	Someone to relax with
	Someone to find a distraction for you
	Someone who needs/loves you
	Scale value social support
**Exposition**	
	Use of sanitary cleaners
	Use of fabric softeners
	Use of floor cleaners
	Use of textile protectants
	Use of air sprays
	Use of fragrances and flavourings
	Use of sunscreen
	Use of body wash/shower gel
	Use of body lotions and creams
	Use of deodorant
	Use of shampoo
	Use of hair styling products
	Use of perfumes
	Use of nail polish
	Use of eye make up
	Use of face make up
**Standard variables of GerES V (if not covered under previous dimensions, Murawski et al. 2020)**	
	Municipality Size
	East Germany/West Germany (Berlin divided)
**Biological variables**	
	Size
	Weight
	BMI (Kromeyer-Hauschild)
	Body surface (Mosteller, 1987)
	Body surface (Du Bois & Du Bois, 1989)
	Creatinine

Figure S1 *CIT analysis of metabolites urine concentration in 3-5-year-olds*

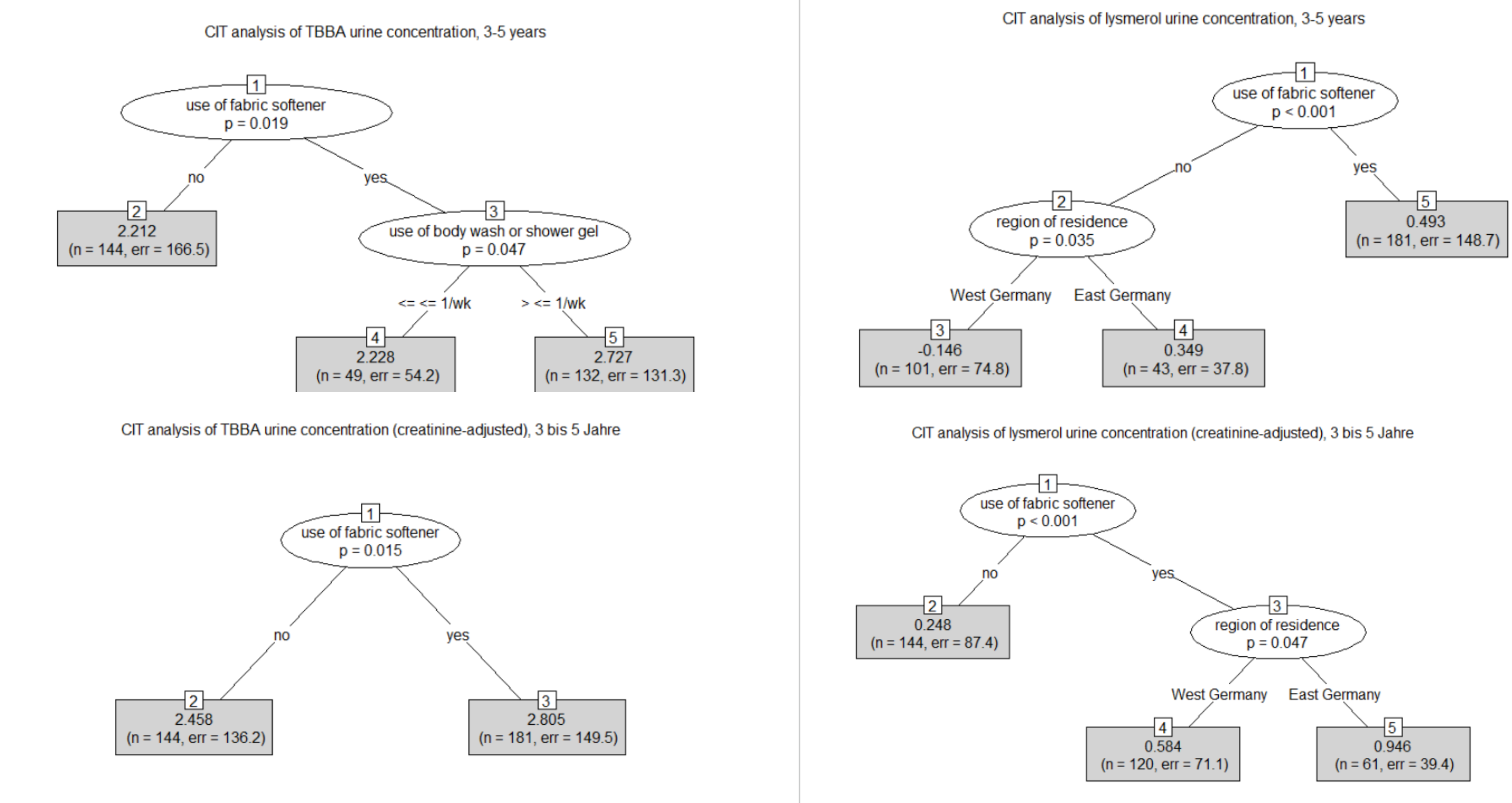

Figure S2 *CIT analysis of metabolites urine concentration in 14-17-year-olds*

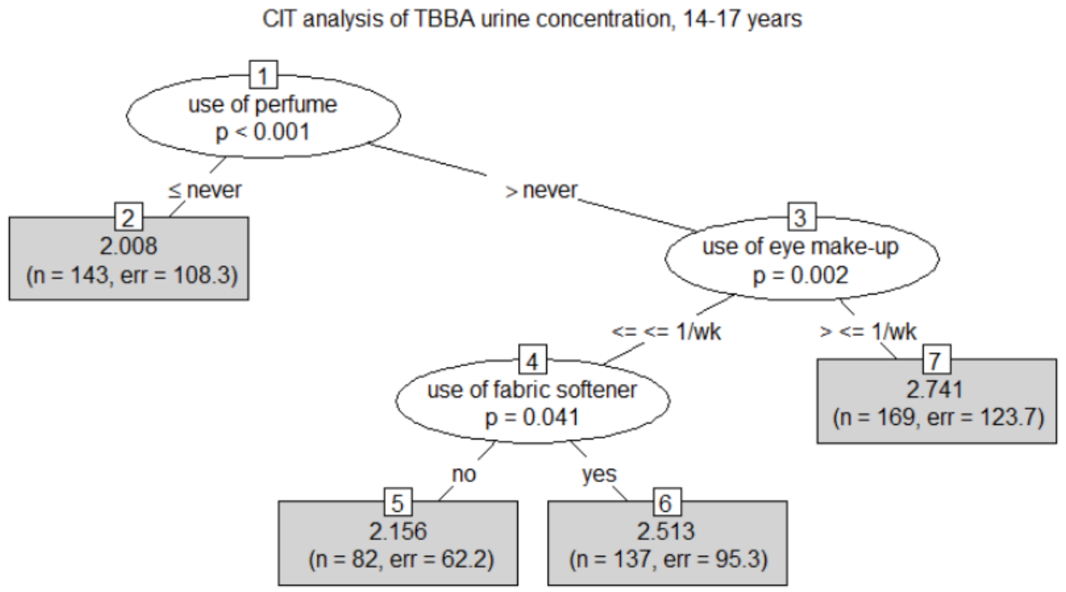

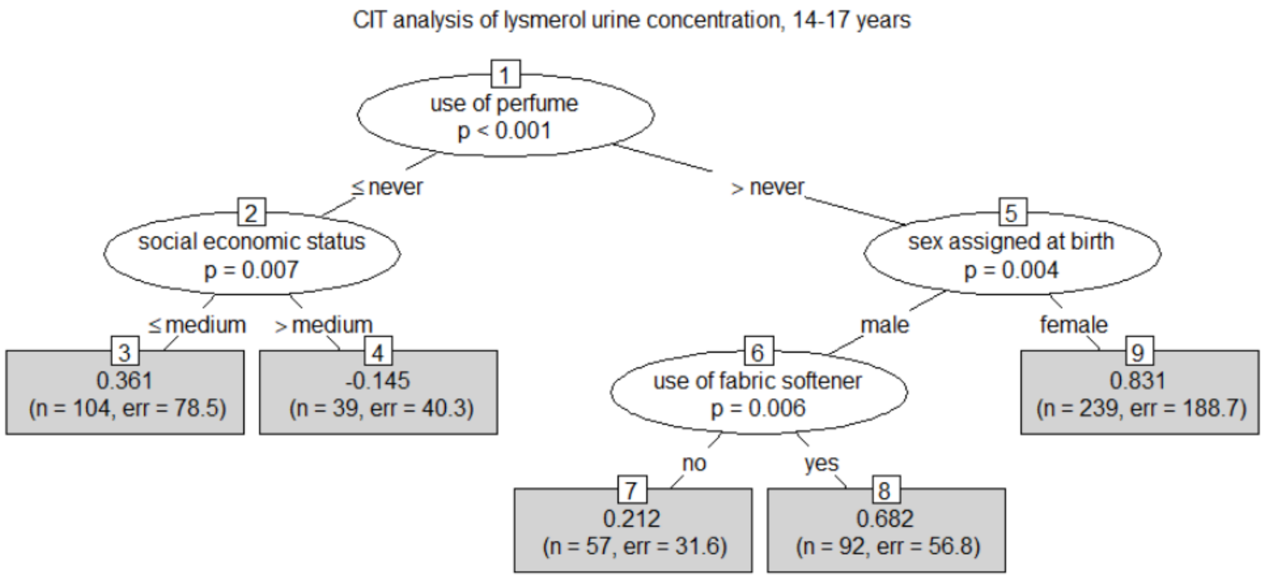

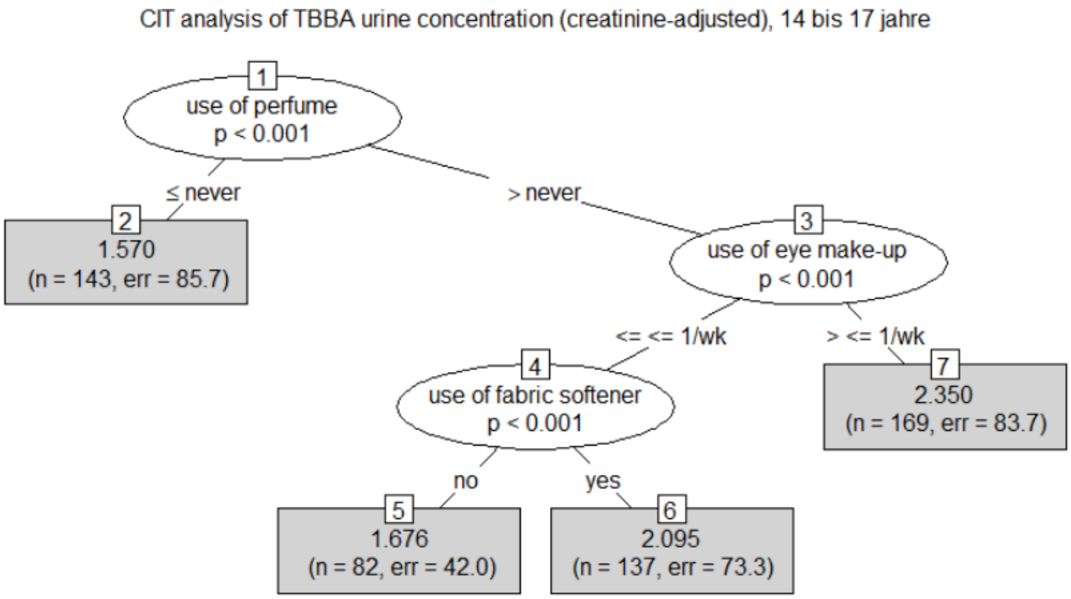
CIT analysis of TBBA urine concentration (creatinine-adjusted), 14 bis 17 jahre
1
use of perfume
p < 0.001
≤ never
> never
2
1.570
(n = 143, err = 85.7)
3
use of eye make-up
p < 0.001
<= <= 1/wk
> <= 1/wk
4
use of fabric softener
p < 0.001
7
2.350
(n = 169, err = 83.7)
no
yes
5
1.676
(n = 82, err = 42.0)
6
2.095
(n = 137, err = 73.3)

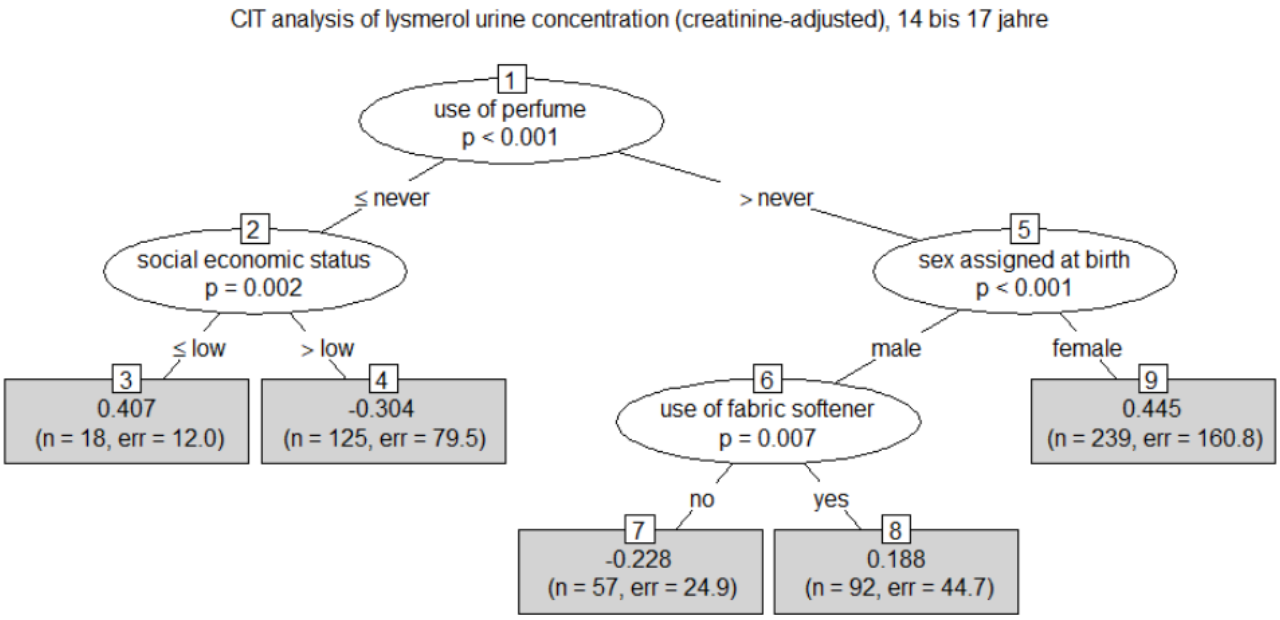
CIT analysis of lysmerol urine concentration (creatinine-adjusted), 14 bis 17 jahre
1
use of perfume
p < 0.001
≤ never
> never
2
social economic status
p = 0.002
5
sex assigned at birth
p < 0.001
≤ low
> low
male
female
3
0.407
(n = 18, err = 12.0)
4
-0.304
(n = 125, err = 79.5)
6
use of fabric softener
p = 0.007
9
0.445
(n = 239, err = 160.8)
no
yes
7
-0.228
(n = 57, err = 24.9)
8
0.188
(n = 92, err = 44.7)

Table S2 *Results of the logistic regression analysis for the urinary TBBA and lysmerol concentration of GerES V participants.*

	TBBA					*Lysmerol*				
**Predictors**	β	*SE*	*CI*	*Statistic*	*p*	β	*SE*	*CI*	*Statistic*	*p*
(Intercept)	0.99	0.23	0.54 – 1.44	4.33	**<0.001**	-0.67	0.21	-1.07 – -0.26	-3.19	**0.001**
**sex assigned at birth**										
male[a]										
female	0.16	0.05	0.07 – 0.25	3.54	**<0.001**	0.10	0.04	0.02 – 0.18	2.43	**0.015**
**age group**										
3-5 years[a]										
6-10 years	-0.40	0.06	-0.52 – -0.29	-6.91	**<0.001**	-0.15	0.05	-0.26 – -0.05	-2.84	**0.005**
11-13 years	-0.67	0.07	-0.80 – -0.54	-10.21	**<0.001**	-0.33	0.06	-0.45 – -0.21	-5.47	**<0.001**
14-17 years	-0.88	0.08	-1.03 – -0.73	-11.61	**<0.001**	-0.45	0.07	-0.58 – -0.31	-6.46	**<0.001**
**use of fabric softener**										
no[a]										
yes	0.23	0.04	0.15 – 0.31	5.48	**<0.001**	0.26	0.04	0.19 – 0.33	6.88	**<0.001**
**use of perfume**										
never[a]										
up to once per week	0.08	0.05	-0.02 – 0.19	1.55	0.121	0.07	0.05	-0.03 – 0.16	1.41	0.159
more than once per week	0.30	0.06	0.18 – 0.43	4.69	**<0.001**	0.23	0.06	0.12 – 0.35	3.94	**<0.001**
**use of air freshener**										
no[a]										
yes	0.04	0.04	-0.04 – 0.13	0.98	0.327	0.06	0.04	-0.02 – 0.14	1.37	0.171
**use of body wash or shower gel**										
never[a]										
up to once per week	0.05	0.15	-0.25 – 0.36	0.35	0.728	-0.09	0.14	-0.36 – 0.19	-0.61	0.545
more than once per week	0.28	0.15	-0.01 – 0.58	1.88	0.061	0.13	0.14	-0.14 – 0.39	0.92	0.360
**use of body lotion**										
never[a]										

less than once per week	0.07	0.06	-0.05 – 0.18	1.17	0.242	0.01	0.05	-0.10 – 0.11	0.11	0.915
once per week	0.08	0.06	-0.04 – 0.20	1.24	0.215	0.06	0.06	-0.05 – 0.17	1.11	0.269
more than once per week	0.12	0.05	0.01 – 0.22	2.20	**0.028**	0.05	0.05	-0.04 – 0.15	1.07	0.287
**region of residence**										
former West Germany[a]										
former East Germany	0.02	0.04	-0.06 – 0.10	0.52	0.603	0.15	0.04	0.08 – 0.23	3.97	**<0.001**
**use of eye make-up**										
never[a]										
up to once per week	-0.09	0.08	-0.25 – 0.06	-1.20	0.231	-0.01	0.07	-0.15 – 0.14	-0.09	0.926
more than once per week	0.18	0.08	0.03 – 0.33	2.30	**0.022**	0.16	0.07	0.03 – 0.30	2.32	**0.021**
**use of floor cleaners**										
no[a]										
yes	0.13	0.04	0.05 – 0.20	3.33	**0.001**	0.04	0.04	-0.03 – 0.11	1.11	0.268
**highest professional qualification**										
foreign [a]										
no qualification (yet)	0.35	0.16	0.03 – 0.67	2.16	**0.031**	0.33	0.15	0.04 – 0.62	2.21	**0.027**
vocational training	0.40	0.15	0.11 – 0.70	2.66	**0.008**	0.31	0.14	0.05 – 0.58	2.30	**0.022**
graduate degree	0.37	0.16	0.06 – 0.68	2.35	**0.019**	0.28	0.14	-0.00 – 0.56	1.94	0.053
**community size**										
rural[a]										
small town	-0.05	0.05	-0.16 – 0.05	-1.00	0.317	-0.02	0.05	-0.11 – 0.08	-0.37	0.714
medium town	-0.05	0.05	-0.15 – 0.06	-0.86	0.389	-0.03	0.05	-0.13 – 0.06	-0.67	0.500
metropolitan	-0.11	0.06	-0.22 – 0.01	-1.78	0.075	-0.07	0.05	-0.17 – 0.04	-1.22	0.223
**social economic status**										
low[a]										
medium	-0.07	0.07	-0.20 – 0.06	-1.09	0.276	-0.24	0.06	-0.36 – -0.13	-4.06	**<0.001**
high	-0.08	0.08	-0.24 – 0.08	-0.95	0.342	-0.35	0.07	-0.50 – -0.21	-4.81	**<0.001**
**use of fragrances**										
no[a]										
yes	0.01	0.04	-0.07 – 0.09	0.18	0.860	0.02	0.04	-0.05 – 0.09	0.62	0.536
Observations	2047					2047				
R^2 / R^2 adjusted	0.247 / 0.237					0.272 / 0.262				

a: Reference group; β: Unstandardized regression coefficient; SE: Standard Error; CI Confidence Intervall; SES: Socioeconomic status; p- values in bold indicate statistically significant differences on the 95% confidence limit.

Table S2 *Results of the logistic regression analysis for the creatinine-normalized urinary TBBA and lysmerol concentration of GerES V participants.*

	TBBA					lysmerol				
**Predictors**	***β***	**SE**	**CI**	**Statistic**	**p**	***β***	**SE**	**CI**	**Statistic**	**p**
(Intercept)	1.83	0.22	1.39 – 2.27	8.19	**<0.001**	0.11	0.20	-0.29 – 0.51	0.54	0.588
**sex assigned at birth**										
male[a]										
female	0.18	0.04	0.10 – 0.27	4.11	**<0.001**	0.13	0.04	0.05 – 0.21	3.28	**0.001**
**age group**										
3-5 years[a]										
6-10 years	-0.51	0.06	-0.62 – -0.40	-9.02	**<0.001**	-0.28	0.05	-0.38 – -0.18	-5.50	**<0.001**
11-13 years	-0.76	0.06	-0.88 – -0.64	-12.17	**<0.001**	-0.46	0.06	-0.57 – -0.35	-8.13	**<0.001**
14-17 years	-0.97	0.07	-1.11 – -0.83	-13.71	**<0.001**	-0.59	0.06	-0.72 – -0.47	-9.29	**<0.001**
**use of fabric softener**										
no[a]										
yes	0.24	0.04	0.16 – 0.32	5.79	**<0.001**	0.27	0.04	0.20 – 0.34	7.33	**<0.001**
**use of perfume**										
never[a]										
up to once per week	0.08	0.05	-0.03 – 0.18	1.48	0.140	0.06	0.05	-0.03 – 0.15	1.30	0.193
more than once per week	0.28	0.06	0.16 – 0.40	4.40	**<0.001**	0.21	0.06	0.09 – 0.32	3.58	**<0.001**
**use of air freshener**										
no[a]										
yes	0.05	0.04	-0.03 – 0.14	1.24	0.214	0.06	0.04	-0.01 – 0.14	1.60	0.109
**use of body wash or shower gel**										
never[a]										

up to once per week	0.06	0.15	-0.24 – 0.35	0.37	0.715	-0.08	0.14	-0.35 – 0.19	-0.57	0.568
more than once per week	0.28	0.15	-0.01 – 0.57	1.89	0.059	0.12	0.13	-0.14 – 0.39	0.92	0.357
**use of body lotion**										
never[a]										
less than once per week	0.07	0.06	-0.04 – 0.19	1.32	0.188	0.01	0.05	-0.09 – 0.11	0.26	0.796
once per week	0.08	0.06	-0.04 – 0.20	1.35	0.178	0.07	0.06	-0.04 – 0.18	1.26	0.208
more than once per week	0.14	0.05	0.03 – 0.24	2.59	**0.010**	0.07	0.05	-0.02 – 0.17	1.52	0.128
**region of residence**										
former West Germany[a]										
former East Germany	0.01	0.04	-0.07 – 0.09	0.30	0.764	0.14	0.04	0.07 – 0.21	3.73	**<0.001**
**use of eye make-up**										
never[a]										
up to once per week	-0.09	0.08	-0.24 – 0.06	-1.18	0.238	-0.01	0.07	-0.15 – 0.13	-0.12	0.901
more than once per week	0.17	0.08	0.02 – 0.32	2.23	**0.026**	0.15	0.07	0.01 – 0.28	2.15	**0.032**
**use of floor cleaners**										
no[a]										
yes	0.12	0.04	0.05 – 0.19	3.15	**0.002**	0.03	0.03	-0.03 – 0.10	0.95	0.342
**highest professional qualification**										
foreign [a]										
no qualification (yet)	0.30	0.16	-0.02 – 0.61	1.85	0.064	0.26	0.15	-0.02 – 0.55	1.81	0.071
vocational training	0.36	0.15	0.07 – 0.65	2.44	**0.015**	0.27	0.13	0.01 – 0.53	2.01	**0.044**
graduate degree	0.34	0.15	0.04 – 0.64	2.19	**0.028**	0.24	0.14	-0.03 – 0.52	1.74	0.083
**community size**										
rural[a]										
small town	-0.07	0.05	-0.17 – 0.04	-1.25	0.211	-0.03	0.05	-0.13 – 0.06	-0.67	0.505
medium town	-0.05	0.05	-0.16 – 0.05	-0.94	0.349	-0.04	0.05	-0.13 – 0.06	-0.81	0.419
metropolitan	-0.10	0.06	-0.22 – 0.01	-1.80	0.072	-0.07	0.05	-0.17 – 0.04	-1.28	0.202
**social economic status**										
low[a]										
medium	-0.06	0.06	-0.18 – 0.07	-0.89	0.374	-0.23	0.06	-0.35 – -0.12	-4.00	**<0.001**
high	-0.08	0.08	-0.23 – 0.08	-0.99	0.324	-0.36	0.07	-0.50 – -0.22	-5.01	**<0.001**
**use of fragrances**										

no[a]										
yes	0.00	0.04	-0.07 – 0.08	0.10	0.921	0.01	0.04	-0.06 – 0.09	0.42	0.677
Observations	2047					2047				
R^2 / R^2 adjusted	0.158 / 0.147					0.152 / 0.142				

a: Reference group; β: Unstandardized regression coefficient; SE: Standard Error; CI Confidence Intervall; SES: Socioeconomic status; p- values in bold indicate statistically significant differences on the 95% confidence limit.
